# *β*_2_-Microglobulin and Neutrophil Gelatinase-Associated Lipocalin, Potential Novel Urine Biomarkers in Periodontitis: A Cross-Sectional Study in Japanese

**DOI:** 10.1155/2019/1394678

**Published:** 2019-03-20

**Authors:** Mayuka Nakajima, Michihiro Hosojima, Koichi Tabeta, Sayuri Miyauchi, Miki Yamada-Hara, Naoki Takahashi, Haruna Miyazawa, Yumi Matsuda-Matsukawa, Keisuke Sato, Noriko Sugita, Yasutaka Komatsu, Tomomi Ishikawa, Kazuhiro Akiishi, Kazuhisa Yamazaki, Kiminori Kato, Akihiko Saito, Hiromasa Yoshie

**Affiliations:** ^1^Division of Periodontology, Department of Oral Biological Science, Niigata University Graduate School of Medical and Dental Science, 2-5274 Gakko-cho, Chuo-ku, Niigata 951-8514, Japan; ^2^Research Unit for Oral-Systemic Connection, Division of Oral Science for Health Promotion, Niigata University Graduate School of Medical and Dental Sciences, 2-5274 Gakko-cho, Chuo-ku, Niigata 951-8514, Japan; ^3^Department of Clinical Nutrition Science, Niigata University Graduate School of Medical and Dental Sciences, Chuo-ku, Niigata 951-8510, Japan; ^4^Research Centre for Advanced Oral Science, Niigata University Graduate School of Medical and Dental Sciences, 2-5274 Gakko-cho, Chuo-ku, Niigata 951-8514, Japan; ^5^Division of Clinical Nephrology and Rheumatology, Niigata University Graduate School of Medical and Dental Sciences, Chuo-ku, Niigata 951-8510, Japan; ^6^Reagent R&D Department, Denka Seiken Co. Ltd., 1359-1, Kagamida, Kigoshi, Gosen-shi, Niigata 959-1695, Japan; ^7^Department of Laboratory Medicine and Clinical Epidemiology for Prevention of Noncommunicable Diseases, Niigata University Graduate School of Medical and Dental Sciences, Chuo-ku, Niigata 951-8510, Japan; ^8^Department of Applied Molecular Medicine, Niigata University Graduate School of Medical and Dental Sciences, Chuo-ku, Niigata 951-8510, Japan

## Abstract

**Objectives:**

Several serum biomarkers have been reported to increase in periodontitis patients as possible mediators linking periodontal inflammation to systemic diseases. However, the relationship between periodontitis and urine biomarkers is still unclear. The aim of this cross-sectional study was to investigate potential urine biomarkers of periodontitis in a Japanese population.

**Materials and Methods:**

This study included 108 male subjects, and microbiological and clinical parameters were evaluated as a periodontitis marker. The correlation between nine urine biomarkers (typically used to diagnose kidney disease) and periodontal parameters was analyzed. Based on the findings, *β*_2_-microglobulin (*β*_2_-MG) and neutrophil gelatinase-associated lipocalin (NGAL) were selected for comparison and multivariate regression analysis, and the Kruskal–Wallis test followed by Bonferroni correction was used to identify differences in their concentrations between the three periodontitis groups (severe, moderate, and no/mild periodontitis).

**Results:**

*β*_2_-MG and NGAL exhibited a significant correlation with clinical parameters of periodontitis. The prevalence of clinical parameters such as bleeding on probing and number of sites with probing depth (PD) ≥ 6 mm were greater in the *β*_2_-MG high group (≥300 *μ*g/g creatinine) than in the normal group (*P*=0.017 and 0.019, respectively). Multivariate regression analysis indicated that the number of sites with PD ≥ 6 mm was independently associated with urine *β*_2_-MG. Moreover, the number of sites with the clinical attachment level (CAL) ≥ 6 mm was greater in the NGAL high group (highest quartile) (*P*=0.041). Multivariate regression analysis showed that the number of sites with CAL ≥ 6 mm was associated independently with urine NGAL. Finally, *β*_2_-MG was significantly higher in the severe periodontitis subjects compared to the no/mild periodontitis subjects.

**Conclusion:**

The significant association between urine *β*_2_-MG or NGAL and periodontitis was revealed. These biomarkers can potentially be used to screen for or diagnose periodontitis. This trial is registered with the UMIN Clinical Trials Registry UMIN000013485.

## 1. Introduction

Periodontitis is a chronic inflammatory disease caused by the host's immune response to subgingival biofilm, resulting in the destruction of the connective tissue and bone that support the teeth. Moreover, inflammation of the local periodontium also induces low-grade systemic inflammation, and this may have a crucial role to play in the increased risk of systemic diseases such as cardiovascular disease (CVD), diabetes, and rheumatoid arthritis associated with periodontitis [1–3]. Although the precise mechanism for this association is still unclear, it has been suggested that periodontopathic bacteria and proinflammatory cytokines produced by the inflamed periodontal tissues can disseminate into the systemic circulation and enhance the inflammatory response of other tissues and organs [4, 5].

At present, periodontitis is diagnosed based on radiographic examination and measurement of clinical parameters such as probing depth (PD), clinical attachment level (CAL), and bleeding on probing (BOP), which can reflect a history of the disease or demonstrate current disease activity. However, no information on systemic biological responses to periodontitis are recorded, and biological markers such as serum biomarkers can act as valuable tools for quantitative detection of the host's response to periodontitis. Several serum biomarkers such as high-sensitivity C-reactive protein (hs-CRP), tumor necrosis factor alpha, interleukin 6, soluble (s) CD14, and antibody titers to periodontopathic bacteria have been reported to reflect periodontitis [6–9]. Moreover, the levels of serum proprotein convertase subtilisin/kexin type 9 (PCSK9) and total bilirubin, both of which exhibit positive or negative relationships with CVD [10], have been reported to increase or decrease in patients with periodontitis [11, 12]. Serum PCSK9 can potentially be used to screen for periodontitis and also evaluate the risk of developing CVD associated with periodontitis [11].

However, in contrast to serum biomarkers, the evidence on the relevance of urine biomarkers in periodontitis is very limited. A previous study reported that urinary albumin excretion increases in patients with periodontitis, and another study conducted in Korea demonstrated that individuals with albuminuria were likely to exhibit a higher prevalence of periodontitis [13, 14]. Prasanna et al. reported that the levels of urine neopterin, which is a marker of cellular immune activation, decrease with periodontal therapy [15]. Urine samples typically contain a lot of biomarkers related to kidney injury, such as albumin (ALB), *β*_2_-microglobulin (*β*_2_-MG), *α*_1_-microglobulin (*α*_1_-MG), N-acetyl-*β*-D-glucosaminidase (NAG), liver-type fatty acid-binding protein (L-FABP), neutrophil gelatinase-associated lipocalin (NGAL), A-megalin (A-Meg), C-megalin (C-Meg), and podocalyxin (PCX). ALB, *β*_2_-MG, *α*_1_-MG, and NAG are classically used to monitor renal impairment or renal tubular dysfunction [16, 17]. L-FABP and NGAL are biomarkers newly recognized in acute kidney injury or CKD [18]. Recently, the measurement of megalin and PCX has been reported to be effective biomarkers to evaluate the severity of CKD [19–23]. However, the relationship between these biomarkers and periodontitis has not been clarified to date. Furthermore, to the best of our knowledge, there are no studies that have attempted to identify the urine biomarkers that are related to periodontitis in a Japanese population.

The aim of the present study was to investigate the relationship between periodontitis and various urine biomarkers typically used to monitor renal function or diagnose kidney diseases. We chose 9 urine biomarkers that have been reported to have an association with kidney injury in this exploratory study.

## 2. Materials and Methods

### 2.1. Study Subjects

**Figure 1:**
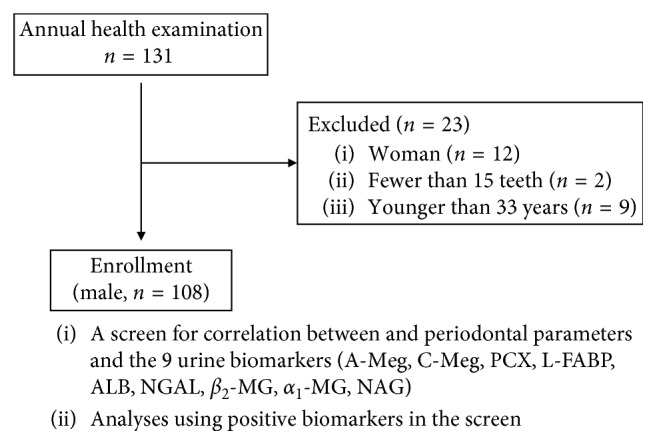
Flow diagram showing the progress of the study. A-Meg, A-megalin; C-Meg, C-megalin; PCX, podocalyxin; L-FABP, liver-type fatty acid binding protein; ALB, albumin; NGAL, neutrophil gelatinase-associated lipocalin; *β*_2_-MG, *β*_2_-microglobulin; *α*_1_-MG, *α*_1_-microglobulin; NAG, *N*-acetyl-*β*-D-glucosaminidase.

This study included 131 industrial employees who had undergone annual health examinations in the city of Niigata, Japan. The same subjects were also enrolled in the study “Prospective observational study of the relationship between periodontal disease and lifestyle-related diseases monitored by atherosclerosis-related biomarkers,” in which the raw data of serum antibody titers against periodontal bacteria and copy number of periodontal bacteria in saliva in addition to examination of periodontitis were obtained and used for the analysis [11]. Individuals with fewer than 15 teeth and the small number of female subjects (*n*=12) were excluded from this study to avoid potential bias. Therefore, the final study sample consisted of 108 males (Figure 1), and the characteristics of the study population are shown in Table 1. The study protocol was approved by the ethics committee of the Niigata University School of Medicine on April 18, 2014 (approval no. 1833), and the study was carried out in accordance with the tenets of the Declaration of Helsinki.

### 2.2. Clinical Measurement

The following clinical outcome variables were measured (described in greater detail previously [11]): PD, CAL, BOP, and O'Leary's plaque control record (PCR). Briefly, probing was performed at six sites around each tooth, and intraexaminer and interexaminer (KT, NS, YK, TH, TO, and NT) calibrations were carried out prior to the measurement. Periodontitis was defined based on the criteria proposed by the Centers for Disease Control and Prevention (CDC) in partnership with the American Academy of Periodontology (AAP) [24]. Subjects with at least two interproximal sites with CAL ≥ 6 mm (in different teeth) and at least one site with PD ≥ 5 mm were diagnosed with “severe periodontitis,” while those with at least two interproximal sites with CAL ≥ 4 mm or at least two interproximal sites with PD ≥ 4 mm (in different teeth) were diagnosed with “moderate periodontitis.” All remaining patients were diagnosed with “no or mild periodontitis.” The smoking status was evaluated in pack-years using a questionnaire [25], and the body mass index was calculated from the subject's height and weight.

### 2.3. Microbiological Assessment for Periodontopathic Bacteria

The microbiological assessments performed for periodontopathic bacteria have been described previously [11]. The copy numbers of total bacteria and periodontopathic bacteria (*Porphyromonas gingivalis*, *Aggregatibacter actinomycetemcomitans*, and *Prevotella intermedia*) in the subgingival plaque were determined by BML, Inc. (Tokyo, Japan) using the PCR-invader method, while antibody titers to the periodontal bacteria (*P. gingivalis*, *A. actinomycetemcomitans*, and *P. intermedia*) were measured using the clinical testing service provided by LEISURE, Inc. (Tokyo, Japan).

### 2.4. Laboratory Assessment for Biological Markers

Serum and urine samples were obtained from all subjects after overnight fasting. The urine biomarkers A-Meg, C-Meg, PCX, L-FABP, ALB, NGAL, *β*_2_-MG, *α*_1_-MG, and NAG were measured as described below. A-Meg, C-Meg, and PCX were measured by Denka Seiken Co., Ltd., Tokyo, Japan, as described previously [20, 21], while L-FABP, ALB, NGAL, *β*_2_-MG, *α*_1_-MG, and NAG were quantified using commercial kits (RENAPRO L-FABP test (CMIC HOLDINGS Co., Ltd., Tokyo, Japan), ALB-TIA N SEIKEN (Denka Seiken Co., Ltd.), NGAL test kit (BioPorto Diagnostics A/S, Gentofte, Denmark), BMG-LATEX X1 SEIKEN (Denka Seiken Co., Ltd.), *α*Mi-LATEX SEIKEN (Denka Seiken Co., Ltd.), and N- assay L NAG NITTOBO (Nittobo Medical Co., Ltd., Tokyo, Japan)). The urinary concentrations of each biomarker were normalized to that of creatinine (expressed as/g Cre). The subjects with *β*_2_-MG ≥ 300 *μ*g/g Cre were assigned to the *β*_2_-MG high group, and the highest quartile was defined as the NGAL high group.

The estimated glomerular filtration rate (eGFR) was calculated using the following Japanese equation: eGFR (mL/min/1.73 m^2^) = 194 × serum Cr^−1.094^ × age^−0.287^ (for males).

Serum high-sensitivity C-reactive protein (hs-CRP) levels were measured using the CRP latex (II) immunoturbidimetric assay (Denka Seiken Co., Ltd.), while hemoglobin A1C (HbA1C) (NGSP) and serum creatinine were measured in an accredited facility (ISO15189) of the Department of Clinical Examination, Niigata Association of Occupation Health Inc. (Niigata, Japan).

### 2.5. Statistical Analysis

Linear correlations between the parameters were analyzed using the Spearman's rank correlation coefficient. The normality of the data was examined using the Shapiro–Wilk test, and based on the results, differences in parameters between the *β*_2_-MG high or NGAL high groups and the others were analyzed using a parametric or nonparametric test (Mann–Whitney *U* test). Based on the findings of the Levene's test, the unpaired or Welch's *t*-test was chosen for further parametric analysis. Multivariate regression analysis was performed by the force entry model, in which the concentration of hs-CRP and the number of sites with PD ≥ 6 mm were categorized to the dummy variable from 1 to 4, and the number of sites with CAL ≥ 6 mm was categorized into 1 to 5 consisting of an equal number of subjects to obtain normalized distribution before analysis. The differences in urine biomarker levels between the three periodontitis groups, as described previously, were compared using the Kruskal–Wallis test followed by a Bonferroni correction. The adjustment for multiple comparison was not performed to avoid missing to detect weak relationship (type II error) in the exploratory study [20, 26]. All statistical analyses were performed using two statistical packages, GraphPad Prism ver. 6 (GraphPad Software Inc., La Jolla, CA, USA) and IBM SPSS 25.0 (IBM Inc., Armonk, New York, USA). The level of significance was set at *P* < 0.05 (indicated using ^*∗*^ for *P* < 0.05 and ^*∗∗*^ for *P* < 0.01).

## 3. Results

### 3.1. Correlation between Urine Biomarkers and Microbiological/Clinical Parameters Related to Periodontitis

The Spearman's correlation coefficient was used to evaluate the association between the urine biomarkers and various microbiological or clinical parameters of periodontitis. The results showed that, of all the urine biomarkers examined, only *β*_2_-MG, *α*_1_-MG, and NGAL were significantly correlated with the clinical parameters. The remaining biomarkers, including A-Meg, C-Meg, PCX, L-FABP, ALB, and NAG, exhibited no such correlation (data not shown). The concentrations of *β*_2_-MG/Cre and *α*_1_-MG/Cre were correlated with the mean PD, mean CAL, and number of sites with CAL = 4 or 5 mm. The concentration of NGAL/Cre was correlated with the number of sites with PD ≥ 6 mm, CAL ≥ 4 mm, CAL = 4 or 5 mm, or CAL ≥ 6 mm. The level of NGAL was also correlated with age, which was one of the potential confounding factors that could influence the levels of urine biomarkers (Table 2). Based on the positive results in the correlation analysis, *β*_2_-MG, *α*_1_-MG, and NGAL were selected for further analysis. However, no significant association was observed between *α*_1_-MG and the periodontal parameters (data not shown), and therefore, only the results of the *β*_2_-MG and NGAL analyses have been reported below.

### 3.2. Association between *β*_2_-MG and Periodontal Parameters

Upon comparison of periodontal parameters between the *β*_2_-MG high (*β*_2_-MG ≥ 300 *μ*g/g Cre) and the normal groups, the *β*_2_-MG high group was seen to exhibit a greater copy number of *Prevotella intermedia* and total bacteria in the subgingival plaque (*P*=0.033 and 0.013, respectively). Furthermore, clinical parameters such as O'Leary's PCR (%), BOP (%), and number of sites with PD ≥ 4 mm, PD = 4 or 5 mm, or PD ≥ 6 mm were significantly greater in the *β*_2_-MG high group compared to the normal group (*P*=0.014, 0.017, 0.016, 0.011, and0.019, respectively). The smoking status (pack-years) was higher in the *β*_2_-MG high group (*P*=0.048), which could be a potential confounder having an effect on *β*_2_-MG levels of the subjects (Table 3) [27].

Multivariate regression analysis was performed to determine the impact of various factors on *β*_2_-MG. The factors selected included potential confounders (i.e., age, smoking status, BMI, eGFR, HbA1C, and hs-CRP) and variables that had exhibited an increase in the *β*_2_-MG high group (BOP, total bacteria in the subgingival plaque, and the number of sites with PD ≥ 6 mm). Of these, the number of sites with PD ≥ 6 mm (i.e., sites with severe periodontitis) was found to be the strongest predictor of *β*_2_-MG (Table 4).

### 3.3. Association between NGAL and Periodontal Parameters

As the NGAL levels were seen to be <21.7 *μ*g/g Cre in all study participants, the threshold concentration of NGAL that contributed to the destruction of periodontal tissue in an incremental manner (data not shown) was examined. The highest quartile (NGAL ≥ 0.49 *μ*g/g Cre) was defined as the NGAL high group, and comparisons of periodontal parameters between this group and all others are shown in Table 5. No significant differences in the potential confounders were observed between the groups. The NGAL high group exhibited a higher copy number of *Porphyromonas gingivalis* in the subgingival plaque (*P*=0.018). Among the clinical parameters, only the number of sites with CAL ≥ 6 mm was seen to be increased in the NGAL high group (*P*=0.041), while no differences in the number of sites with PD ≥ 6 mm were observed between the groups (*P*=0.106).

Multivariate regression analysis with NGAL as a dependent factor was carried out and, once again, the variables selected included potential confounders and those that had exhibited an increase in the NGAL high group (*Porphyromonas gingivalis* in the subgingival plaque and number of sites with CAL ≥ 6 mm). Of these, the number of sites with CAL ≥ 6 mm (cumulative severe destruction of the periodontium) was found to be the strongest predictor of NGAL (Table 6).

### 3.4. Comparison of the Concentration of Urine Biomarkers between Periodontitis Groups

**Figure 2:**
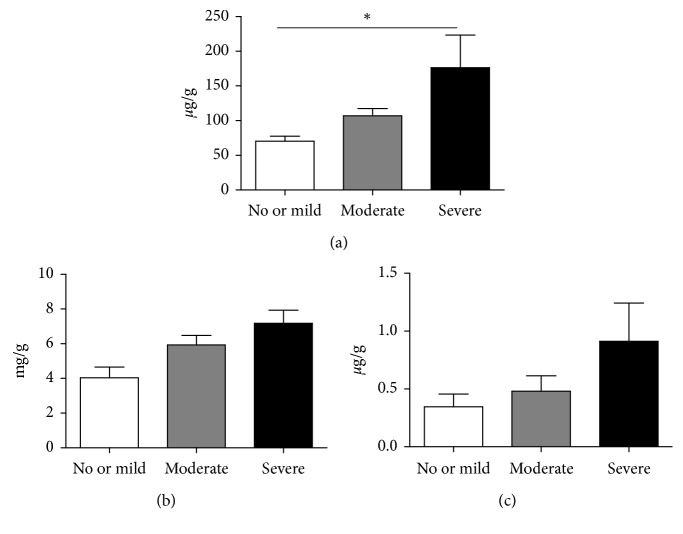
Comparison of the concentration of urine biomarkers among groups classified by the severity of periodontitis. (a) *β*_2_-MG/Cre. (b) *α*_1_-MG/Cre. (c) NGAL/Cre. Results are shown as mean ± standard error of the mean (SEM). The whole subjects were classified by the severity of periodontitis according to the criteria of the CDC/AAP as described in Materials and Methods. The number of subjects in each group was as follows. Severe periodontitis: *n*=39; moderate periodontitis: *n*=60; no or mild periodontitis: *n*=9. The significance of differences of urine biomarkers was determined by Kruskal–Wallis test followed by Bonferroni correction. ^*∗*^Values statistically significant at ^*∗*^*P* < 0.017. NGAL, neutrophil gelatinase-associated lipocalin; *β*_2_-MG, *β*_2_-microglobulin; *α*_1_-MG, *α*_1_-microglobulin; Cre, creatinine.

The study participants were assigned to groups based on the severity of the periodontitis exhibited (in accordance with the criteria proposed by the CDC/AAP) [24]. The differences in *β*_2_-MG, *α*_1_-MG, and NGAL levels between the groups were analyzed using the Kruskal–Wallis test followed by a Bonferroni correction. The results showed that the urinary concentrations of all the 3 biomarkers tended to increase with the severity of periodontitis, and the *β*_2_-MG value was significantly higher in the severe periodontitis group compared to the no or mild periodontitis groups (*P*=0.009) (Figure 2).

## 4. Discussion

To the best of our knowledge, this was the first cross-sectional study that examined the relationship between periodontitis and various urine biomarkers commonly used to monitor or diagnose kidney function in a Japanese population. A previous study reported that antibody titers to *P. gingivalis* correlate with albuminuria in nonobese Japanese type 2 diabetic patients; however, the periodontal status of these patients is not defined [28]. Among the urine biomarkers, *β*_2_-MG, *α*_1_-MG, and NGAL were seen to be positively correlated with the clinical periodontal status. Although, urinary albumin was reported to have a relationship with periodontitis [14], no association with parameters of periodontitis was observed in this study. This conflict could be due to differences in the severity of albuminuria, criteria for periodontitis, and ethnicity of subjects.

*β*_2_-MG and *α*_1_-MG are low-molecular-weight proteins (27 and 11.8 kDa, respectively), with the former being produced by all cells expressing major histocompatibility complex class I antigen and the latter being synthesized mainly by the liver and existing in various body fluids [29, 30]. The proteins are readily filtered through the glomerulus in a healthy kidney, and approximately 99% is reabsorbed and catabolized by the proximal tubular cells. Therefore, increased *β*_2_-MG or *α*_1_-MG excretion in urine has been reported to indicate early signs of renal tubular dysfunction [16, 17, 31, 32]. The concentration of urine *β*_2_-MG is also known to increase during various inflammatory conditions or viral infections independent of kidney injury [33, 34]. In the current study, higher inflammatory activity was observed in the PD and BOP (indicating severe periodontitis) of individuals included in the high *β*_2_-MG group compared to those in the normal *β*_2_-MG group. Furthermore, multivariate regression analysis indicated that severe periodontitis was independently associated with the urine concentration of *β*_2_-MG.

Previous studies have reported increased concentrations of *β*_2_-MG in the gingival crevicular fluid, saliva, and serum of patients with periodontitis [35–37]. However, the mechanism by which periodontitis affects the urinary levels of *β*_2_-MG is still unclear, and future studies should also focus on examining the levels of *β*_2_-MG in gingival tissues. High concentrations of *β*_2_-MG in inflamed periodontal tissues may disseminate into the systemic circulation and be excreted through the urine, thus exhibiting increased levels. On the contrary, due to the bidirectional relationship between renal function and periodontitis [38, 39], urine *β*_2_-MG possibly increased along with renal dysfunction and associated with periodontitis. However, the participants of the current study were not diagnosed as renal dysfunction. Further studies are necessary to better understand the mechanism underlying increased urinary concentrations of *β*_2_-MG in patients with periodontitis.

The results of this study showed an association between the urinary concentration of NGAL and the severity of periodontitis. As increased concentrations of NGAL in the renal tubules are typically associated with kidney injuries, it can be considered as a sensitive biomarker for nephropathy [18, 40, 41]. Interestingly, Morelli et al. reported higher salivary levels of NGAL in patients with severe periodontitis [42]. This biomarker is mainly secreted by neutrophils in a healthy state [43] and is, therefore, also thought to play a role in the regulation of inflammation and antimicrobial defense [44–46]. Additionally, NGAL is a pleiotropic protein that stabilizes matrix metalloproteinase 9 [47, 48], which is a catabolic enzyme for type IV collagen, resulting in acceleration of the periodontium destruction [49]. Hence, NGAL may potentially play a role in the pathogenesis of periodontitis, although no information on the gingival NGAL levels of the participants included in the current study were available.

The use of biological markers for the diagnosis and treatment of periodontitis has the following clinical benefits: (1) quantitative detection of the systemic biological response that reflects the periodontal status, (2) allows assessment of the risk of systemic diseases related to periodontitis, and is (3) potentially useful for screening for periodontitis without the need for clinical examination by dentists. Urine samples are frequently used for health examinations and can be collected without causing pain or discomfort. Moreover, urinary levels of *β*_2_-MG and NGAL have been used clinically to diagnose kidney injuries. Therefore, these markers can be quite easily utilized clinically for the screening of periodontitis by medical doctors.

The limitations of this study include the cross-sectional study design, the small number of male subjects included, and the absence of multiple adjustments. Hence, future studies should use longitudinal and interventional designs and larger sample sizes in order to confirm the exact association between periodontitis and *β*_2_-MG or NGAL levels.

It is essential to bear in mind that the participants included in the current study had been examined previously in another study focusing on the relationship between serum biological markers of atherosclerotic disease and periodontitis [11]. This manuscript was prepared with careful consideration of the ethical issues in research [50, 51]. Firstly, all classifications of subjects and statistical analyses were carried out separately, as the focus and aims of each study were distinct. Secondly, the samples for analysis were different, with the current study using urine and the previous study evaluating serum. The results of the two studies have been presented separately as their combination produced findings that were too vast to be discussed in one article.

## 5. Conclusion

In conclusion, within the limitations of this study, the findings suggest that urinary *β*_2_-MG and NGAL are potential biomarkers associated with periodontal parameters. However, the underlying mechanism by which periodontitis increases the urinary levels of these markers as well as their efficacy in the diagnosis or screening of periodontitis are still unclear. Further studies are needed to determine these proteins as a certain biological marker of periodontitis.

## Figures and Tables

**Table 1:** Characteristics of the study population.

	Mean ± SD	Min–Max
Age (years)	50.1 ± 8.6	33.0–68.0
Smoking status (pack-years)	11.5 ± 16.7	0.0–80.0
BMI	23.5 ± 2.8	16.6–32.4
eGFR (ml/min/1.73 m^2^)	81.4 ± 13.0	53.5–125.7
HbA1C (%)	5.4 ± 0.4	3.8–6.9
Parameters related to periodontitis		
O'Leary's PCR (%)	47.0 ± 22.6	0.0–99.0
BOP (%)	14.6 ± 14.0	0.0–73.8
Mean PD (mm)	2.5 ± 0.6	1.2–4.8
PD ≥ 4 mm (number of sites)	17.3 ± 22.3	0.0–114.0
PD 4, 5 mm (number of sites)	14.2 ± 16.4	0.0–82.0
PD ≥ 6 mm (number of sites)	3.1 ± 7.7	0.0–51.0
Mean CAL (mm)	2.7 ± 0.7	1.2–5.4
CAL ≥ 4 mm (number of sites)	28.6 ± 26.0	0.0–114.0
CAL 4, 5 mm (number of sites)	23.8 ± 19.5	0.0–91.0
CAL ≥ 6 mm (number of sites)	4.8 ± 9.4	0.0–58.0

Data are shown as mean ± SD and the range (minimal to maximum). BMI, body mass index; eGFR, estimated glomerular filtration rate; HbA1C, hemoglobin A1C (NGSP); O'Leary's PCR, O'Leary's plaque control record; BOP, bleeding on probing; PD, probing depth; CAL, clinical attachment level.

**Table 2:** Correlation of urine biological markers with microbiological/clinical parameters related to periodontitis.

	NGAL/Cre *ρ* (*P* value)	*β*_2_-MG/Cre *ρ* (*P* value)	*α*_1_-MG/Cre *ρ* (*P* value)
Age (years)	0.235 (0.015)^*∗*^	0.086 (0.378)	0.137 (0.158)
Subgingival plaque (copy)			
*P. gingivalis*	0.185 (0.055)	0.100 (0.305)	0.092 (0.344)
*P. intermedia*	–0.107 (0.271)	0.158 (0.102)	0.183 (0.057)
*A. actinomycetemcomitans*	–0.106 (0.273)	–0.040 (0.683)	–0.102 (0.296)
Total bacteria	0.043 (0.657)	0.111 (0.253)	0.160 (0.097)
Antibody titer (unit)			
*P. gingivalis*	0.110 (0.255)	0.082 (0.400)	–0.012 (0.904)
*P. intermedia*	–0.015 (0.876)	0.021 (0.827)	–0.214 (0.026)^*∗*^
*A. actinomycetemcomitans*	–0.003 (0.972)	0.065 (0.505)	0.053 (0.588)
Parameters related to periodontitis			
O'Leary's PCR (%)	0.089 (0.362)	0.043 (0.659)	–0.054 (0.578)
BOP (%)	0.056 (0.564)	–0.006 (0.954)	–0.111 (0.251)
Mean PD (mm)	0.037 (0.706)	0.193 (0.045)^*∗*^	0.271 (0.005)^*∗∗*^
PD ≥ 4 mm (number of sites)	0.124 (0.201)	0.118 (0.223)	0.158 (0.102)
PD 4, 5 mm (number of sites)	0.091 (0.349)	0.119 (0.221)	0.171 (0.078)
PD ≥ 6 mm (number of sites)	0.211 (0.029)^*∗*^	0.126 (0.193)	0.105 (0.279)
Mean CAL (mm)	0.129 (0.182)	0.248 (0.010)^*∗*^	0.266 (0.005)^*∗∗*^
CAL ≥ 4 mm (number of sites)	0.214 (0.026)^*∗*^	0.182 (0.059)	0.184 (0.056)
CAL 4, 5 mm (number of sites)	0.193 (0.045)^*∗*^	0.193 (0.046)^*∗*^	0.192 (0.047)^*∗*^
CAL ≥ 6 mm (number of sites)	0.256 (0.007)^*∗∗*^	0.123 (0.205)	0.097 (0.317)

Spearman's rank correlation coefficient was performed for statistical analysis. The statistically significant values are indicated by ^*∗*^*P* < 0.05, ^*∗∗*^*P* < 0.01. NGAL, neutrophil gelatinase-associated lipocalin; *β*_2_-MG, *β*_2_-microglobulin; *α*_1_-MG, *α*_1_-microglobulin; Cre, creatinine; *P. gingivalis*, *Porphyromonas gingivalis*; *P. intermedia*, *Prevotella intermedia*; *A. actinomycetemcomitans*, *Aggregatibacter actinomycetemcomitans*; O'Leary's PCR, O'Leary's plaque control record; BOP, bleeding on probing; PD, probing depth; CAL, clinical attachment level.

**Table 3:** Comparison of microbiological/clinical parameters related to periodontitis between *β*_2_-MG high and normal groups.

Group	*β*_2_-MG/Cre(*μ*g/g)		
	Normal	High	*P* value
Number of subjects	102	6	
Age (years)	50.2 ± 8.5	48.7 ± 10.4	0.679
Smoking status (pack-years)	10.8 ± 16.5	22.8 ± 18.1	0.048^*∗*^
BMI	23.5 ± 2.9	22.3 ± 1.9	0.304
eGFR (ml/min/1.73 m^2^)	81.5 ± 13.0	80.9 ± 14.0	0.913
HbA1C (%)	5.4 ± 0.3	5.7 ± 0.8	0.866
Subgingival plaque (copy)			
*P. gingivalis*	3.5E + 5 ± 1.2E + 6	4.5E + 5 ± 8.7E + 5	0.216
*P. intermedia*	9.7E + 4 ± 3.3E + 5	1.0E + 5 ± 8.8E + 4	0.033^*∗*^
*A. actinomycetemcomitans*	1.1E + 3 ± 7.5E + 3	8.0E + 3 ± 2.0E + 4	0.205
Total bacteria	2.9E + 7 ± 5.8E + 7	7.1E + 7 ± 5.1E + 7	0.013^*∗*^
Antibody titer (unit)			
*P. gingivalis*	8.4 ± 10.9	11.5 ± 14.9	0.648
*P. intermedia*	0.3 ± 0.7	0.2 ± 0.3	0.841
*A. actinomycetemcomitans*	0.5 ± 1.3	1.0 ± 1.1	0.227
Parameters related to periodontitis			
O'Leary's PCR (%)	45.9 ± 22.7	66.7 ± 5.2	0.014^*∗*^
BOP (%)	13.7 ± 13.4	28.9 ± 16.8	0.017^*∗*^
Mean PD (mm)	2.5 ± 0.6	3.0 ± 0.8	0.105
PD ≥ 4 mm (number of sites)	15.4 ± 20.0	50.0 ± 35.4	0.016^*∗*^
PD 4, 5 mm (number of sites)	12.5 ± 13.7	43.0 ± 30.0	0.011^*∗*^
PD ≥ 6 mm (number of sites)	2.9 ± 7.7	7.0 ± 6.5	0.019^*∗*^
Mean CAL (mm)	2.7 ± 0.7	3.2 ± 0.9	0.155
CAL ≥ 4 mm (number of sites)	26.8 ± 23.8	59.5 ± 42.0	0.069
CAL 4, 5 mm (number of sites)	22.5 ± 17.8	46.7 ± 32.7	0.068
CAL ≥ 6 mm (number of sites)	4.3 ± 8.9	12.8 ± 14.3	0.060

Results are shown as mean ± SD. Age, BMI, and eGFR: unpaired *t*-test. Other parameters: Mann–Whitney *U* test. Statistical significance is indicated by ^*∗*^*P* < 0.05. *β*_2_-MG, *β*_2_-microglobulin; Cre, creatinine; BMI, body mass index; eGFR, estimated glomerular filtration rate; HbA1C, hemoglobin A1C (NGSP); *P. gingivalis*, *Porphyromonas gingivalis*; *P. intermedia*, *Prevotella intermedia*; *A. actinomycetemcomitans*, *Aggregatibacter actinomycetemcomitans*; O'Leary's PCR, O'Leary's plaque control record; BOP, bleeding on probing; PD, probing depth; CAL, clinical attachment level.

**Table 4:** Multivariate regression analysis of the associations between *β*_2_-MG and variables.

	Standardized *β*	*t*	*P* value
Age (years)	0.174	1.783	0.078
Smoking status (pack-years)	0.021	0.200	0.842
BMI	−0.174	−1.605	0.112
eGFR (ml/min/1.73 m^2^)	0.049	0.462	0.645
HbA1C (%)	0.040	0.392	0.696
Hs-CRP	−0.032	−0.317	0.752
BOP (%)	−0.060	−0.470	0.639
Total bacteria in the subgingival plaque (copy)	−0.098	−0.851	0.397
PD ≥ 6 mm (number of sites)	0.280	2.234	0.028^*∗*^

Multivariate regression analysis was performed by the force entry model. Age, smoking status, BMI, eGFR, HbA1C, hs-CRP, BOP, total bacteria in the subgingival plaque, and the number of sites with PD ≥ 6 mm were used as variables. To obtain the normal distribution, the values of total bacteria in the subgingival plaque were used after logarithmic conversion. hs-CRP and the number of sites with PD ≥ 6 mm were converted to an ordinal value as described in Materials and Methods. *R* = 0.355; adjusted *R*^2^=0.045. Statistically significant values are indicated by ^*∗*^*P* < 0.05.

**Table 5:** Comparison of microbiological/clinical parameters related to periodontitis between highest quartile of NGAL and others.

	NGAL/Cre (*μ*g/g)		
	Others	Highest quartile	*P* value
Number of subjects	81	27	
Age (years)	49.5 ± 8.5	51.9 ± 8.6	0.198
Smoking status (pack-years)	11.6 ± 16.5	11.3 ± 17.7	0.987
BMI	23.6 ± 2.9	23.0 ± 2.7	0.307
eGFR	80.9 ± 13.1	82.9 ± 13.0	0.510
HbA1C (%)	5.5 ± 0.4	5.3 ± 0.4	0.169
Subgingival plaque (copy)			
*P. gingivalis*	2.3E + 5 ± 6.5E + 5	7.6E + 5 ± 2.0E + 6	0.018^*∗*^
*P. intermedia*	6.6E + 4 ± 1.5E + 5	1.9E + 5 ± 5.9E + 5	0.514
*A. actinomycetemcomitans*	1.8E + 3 ± 9.9E + 3	3.0E + 2 ± 1.5E + 3	0.617
Total bacteria	2.7E + 7 ± 4.3E + 7	4.4E + 7 ± 9.0E + 7	0.199
Antibody titer (unit)			
*P. gingivalis*	9.2 ± 12.3	6.8 ± 6.3	0.796
*P. intermedia*	0.3 ± 0.7	0.1 ± 0.5	0.305
*A. actinomycetemcomitans*	0.6 ± 1.4	0.5 ± 1.1	0.607
Parameters related to periodontitis			
O'Leary's PCR (%)	45.7 ± 22.9	50.9 ± 21.9	0.354
BOP (%)	14.8 ± 14.2	13.9 ± 13.5	0.777
Mean PD (mm)	2.6 ± 0.6	2.4 ± 0.6	0.500
PD ≥ 4 mm (number of sites)	17.4 ± 24.2	17.1 ± 16.1	0.548
PD 4, 5 mm (number of sites)	14.2 ± 17.2	14.2 ± 13.9	0.784
PD ≥ 6 mm (number of sites)	3.2 ± 8.6	2.9 ± 3.8	0.106
Mean CAL (mm)	2.8 ± 0.7	2.7 ± 0.7	0.640
CAL ≥ 4 mm (number of sites)	27.5 ± 27.6	31.8 ± 20.5	0.093
CAL 4, 5 mm (number of sites)	23 .0 ± 20.5	26.2 ± 16.3	0.151
CAL ≥ 6 mm (number of sites)	4.5 ± 10.2	5.7 ± 6.4	0.041^*∗*^

Results are shown as mean ± SD. Age, BMI, and eGFR: unpaired *t*-test. Other parameters: Mann–Whitney *U* test. Statistical significance is indicated by ^*∗*^*P* < 0.05. NGAL, neutrophil gelatinase-associated lipocalin; Cre, creatinine; BMI, body mass index; eGFR, estimated glomerular filtration rate; HbA1C, hemoglobin A1C (NGSP); *P. gingivalis*, *Porphyromonas gingivalis*; *P. intermedia*, *Prevotella intermedia*; *A. actinomycetemcomitans*, *Aggregatibacter actinomycetemcomitans*; O'Leary's PCR, O'Leary's plaque control record; BOP, bleeding on probing; PD, probing depth; CAL, clinical attachment level.

**Table 6:** Multivariate regression analysis of the associations between NGAL and variables.

	Standardized *β*	*t*	*P* value
Age (years)	0.154	1.550	0.124
Smoking status (pack-years)	−0.084	−0.859	0.393
BMI	−0.109	−1.020	0.310
eGFR (ml/min/1.73 m^2^)	0.008	0.073	0.942
HbA1C (%)	−0.057	−0.550	0.583
Hs-CRP	−0.134	−1.355	0.178
BOP (%)	−0.184	−1.571	0.119
*P. gingivalis* in subgingival plaque (copy)	0.008	0.067	0.947
CAL ≥ 6 mm (number of sites)	0.260	2.206	0.030^*∗∗*^

Multivariate regression analysis was performed by the force entry model. Age, smoking status, BMI, eGFR, HbA1C, hs-CRP, BOP, copy number of *P. gingivalis* in the subgingival plaque, and the number of sites with CAL ≥ 6 mm were used as variables. To obtain the normal distribution, the values of *P. gingivalis* in the subgingival plaque were used after logarithmic conversion. hs-CRP and the number of sites with CAL ≥ 6 mm were converted to an ordinal value as described in Materials and Methods. *R* = 0.391; adjusted *R*^2^ = 0.074. ^*∗*^Values statistically significant at *P* < 0.05.

## Data Availability

The data used to support the findings of this study are restricted by the Ethics Committee of the Niigata University School of Medicine in order to protect patients' privacy. The anonymized data are available from the corresponding author for researchers who meet the criteria for access to data.
